# High burden of invasive and non-invasive cancer among women aged 20–49 years: the situation in Geneva, Switzerland

**DOI:** 10.1186/s12905-022-01933-5

**Published:** 2022-08-18

**Authors:** Elisabetta Rapiti, Evelyne Fournier, Robin Schaffar

**Affiliations:** Geneva Cancer Registry, University of Geneva, CMU – Rue Michel Servet 1, 1211 Geneva 4, Switzerland

**Keywords:** Cancer, Epidemiology, Incidence, Sex distribution, Young adults

## Abstract

**Background:**

The pattern of cancer among young adults aged 20–49 years is different than that observed in other age groups, most notably women present higher rates than those observed among men. Estimations of the burden of cancer disease among women of this age group, however, rarely include both invasive and non-invasive disease.

**Methods:**

We calculated incidence rates of invasive and non-invasive cancers for women and men aged 20–49 years and by cancer site for the period 2014–2018 in the canton of Geneva, Switzerland using data from the population-based cancer registry.

**Results:**

Between 2014 and 2018, the incidence rates of invasive and non-invasive cancers among women were 177.6/100,000 and 166.4/100,000, respectively. The rates among men of the same age were 110.6/100,000 and 31.8/100,000, respectively. Just three cancers, breast, thyroid and melanoma accounted for 70% of all invasive cancers among women. In situ cervical cancer represented over 70% of non-invasive disease.

**Conclusion:**

Cancer among women aged 20–49 years is quite frequent. This is primarily a result of increasing risk, increased diagnosis or both and highlights the need for better primary prevention strategies, personalized risk assessment and tailored screening, as well as increased awareness of women and health professionals about health risks in young adults.

## Highlights

- The burden of cancer among people aged 20–49 is unequally distributed between men and women in Geneva.
- Women had 1.6- and five-times higher rates than men respectively for invasive and non-invasive cancers.
- Breast cancer, thyroid cancer and melanomas were the more frequent invasive cancer diagnoses observed among women.
- Cervical cancer accounted for 72% of the non-invasive cancer diagnoses.
- Increased cancer risk and increased detection are the drivers of this finding

## Background

Although overall cancer incidence and cancer mortality are higher in men than in women [1], this pattern is reversed in people aged 20–49 years. Ward et al. describing the cancer occurrence in the USA in their annual report, found unequal risk of cancer between men and women aged 20–49 with estimates of invasive cancer of 115.3/100,000 among men and of 203.3 for women in 2011–2015 [2]. The International Agency for Research on Cancer has recently emphasized the global nature of this finding and highlighted three cancers, breast, thyroid and cervical cancer, as responsible for the higher risk among women and some of the circumstances related to this increased burden [3].

However, the magnitude and the nature of this difference, have not been fully evaluated. Attempts to characterize this finding have only taken into account the occurrence of invasive tumors, and the one exception that considered the impact of non-invasive tumors only assessed in situ breast cancer [2].

In this report we describe the burden of invasive and non-invasive cancer among women aged 20–49 years in the canton of Geneva (Switzerland) between 2014 and 2018. We present incidence rates in women compared with those of men and discuss the underlying reasons and resulting impact on individuals and the health system.

## Patients and methods

We used data from the Geneva cancer registry, which records all the new cancer diagnoses occurring in the canton (500,000 inhabitants). Hospitals, pathology laboratories, and private practitioners are requested to report all cancer cases. We calculated the mean annual age-adjusted incidence rates (per 100,000 European standard population) of invasive and non-invasive cancers (in situ, ICDO codes D00-09) excluding non-melanotic skin tumours for women and men in the age group 20–49 years overall and by cancer site for the period 2014–2018. To identify the main drivers in terms of type of cancer, based on the RARECARE definition for rare cancers, we included in the descriptive table only cancers whose rate was > 6/100,000 in at least one of the two genders [4].

## Results

**Fig. 1:**
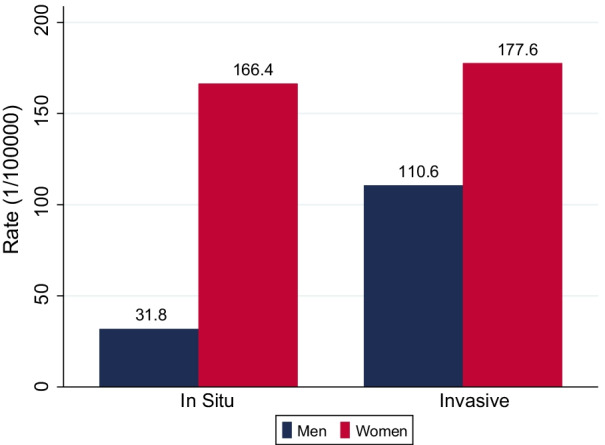
European Age-adjusted incidence rates for in-situ and invasive tumours among women and men aged 20–49. Geneva. 2014–2018

Between 2014 and 2018, 997 women and 595 men aged 20–49 years were diagnosed with an invasive cancer in Geneva, 912 and 172 respectively with a non-invasive cancer. The adjusted incidence rate for invasive cancers was 177.6/100,000 for women and 110.6/100,000 for men; for non-invasive tumors 166.4/100,000 for women and 31.8/100,000 for men, respectively (Fig. 1).

The five most frequent invasive cancers diagnosed among women were breast cancer (n = 475, rate: 88.3), thyroid cancer (n = 126, rate: 23.4), skin melanoma (n = 95, rate: 17.7) colorectal cancer (n = 40, rate: 7.4) and lymphoma Non-Hodgkin (n = 28, 5.2) (Table 1). Among men, the picture was more diverse, with five cancers accounting for 50% of all cancers: we observed melanoma (n = 91, 16.9), testicular cancer (n = 77, 14.5), colorectal cancer (n = 56, 10.4), lymphoma non-Hodgkin (n = 47, 8.7), and thyroid cancer (n = 28, 5.2).

**Table 1:** Incidence data by sex and cancer sites for patients aged 20–49. Geneva. 2014–2018

Cancer site**	Women			Men		
	N	Rate*	95%CI	N	Rate*	95%CI
*Invasive cancers*						
Colorectal	40	7.2	[5.1–9.8]	56	10.4	[7.8–13.5]
Melanoma	95	17.1	[13.8–20.9]	91	16.9	[13.6–20.7]
Breast	475	83.8	[76.5–91.7]	–	–	–
Testis	–	–	–	77	14.5	[11.4–18.1]
Thyroid	126	22.6	[18.8–26.9]	28	5.2	[3.4–7.5]
Non Hodgkin Lymphoma	28	5.2	[3.5–7.6]	47	8.7	[6.4–11.6]
All invasive tumours	*997*	*177.6*	*[166.7–189.0]*	*595*	*110.6*	*[101.8–119.8]*
*Non-invasive cancers*						
Melanoma	134	23.8	[19.9–28.2]	108	20	[16.4–24.1]
Breast	64	11.3	[8.7–14.5]	–	–	–
Cervical	656	121	[111.9–130.7]	–	–	–
All in situ tumours	*912*	*166.4*	*[155.8–177.6]*	*172*	*31.8*	*[27.2–36.9]*

Values in italics are totals

^*^ European Age adjusted incidence rate. 1/100,000

^**^ Only sites with incidence rate > 6/100,000 are presented

In women the most frequent non-invasive cancers were in situ cervical cancer (cervical intraepithelial neoplasia, CIN3 ICDO D06) (n = 656, 121.0), melanoma in situ (n = 134, 23.8) and in situ breast cancer (n = 64, 11.3). In situ melanoma was the only non-invasive cancer among men with a rate greater than 6/100,000 (n = 108, 20.0) (Table 1).

## Discussion

A cancer diagnosis is one of the most disrupting experiences people may have in their lifetime and one of the most complex, time and resource demanding on a health system. A cancer diagnosis for a woman aged 20–49 years, when many are still completing their education, embarking on their professional life and/or starting a family, can have particularly strong social, psychological and health impacts, including those due to the long-term effect of treatment. The most recent cancer data from Geneva show that cancer diagnosis among women aged 20–49 years is quite frequent, in terms of both invasive and non-invasive disease. Compared to men of the same age, women are 60% more likely to be diagnosed with an invasive cancer, and over 5 times more likely to be diagnosed with a non-invasive cancer.

Among young adult women, three cancers (breast cancer, thyroid cancer and melanomas) account for 70% of all diagnoses of invasive cancer and only one type of cancer (in-situ cervical cancer) is responsible of 72% of all non-invasive tumours. The factors that drive to these findings are complex and diverse as are the implications in terms of prevention, diagnosis, treatment, follow up procedure, and prognosis.

Breast cancer ranks first among invasive cancers in this age group and is the leading cause of cancer death among women in general [1]. Here we confirm that the incidence rate of breast cancer among young women is still high, as we described, among the first, in 2012 [5]. Most organisations in Europe do not recommend mammography screening for women less than 50 [6], while in the US, the recommendations vary greatly, with the American Cancer Society recommending annual screening between the ages of 45 and 54 with the option of starting annual screening between 40 and 44 [7]. The recent conclusions of the UK Age trial that lowering the age limit for mammography screening from 50 to 40 could potentially reduce breast cancer mortality in this age group [8], although promising, were challenged from other authors, implying that screening at this young age may come with an increased risk of false-positives, overdiagnosis and long-term adverse effects [9–12].

For this reason, and because risk factors are poorly known and the cancer often has different characteristics and a worse prognosis [13], access to early diagnosis, timely and high-quality treatment as well as survivor care is even more important to improve health outcomes and quality of life in these women. Most important is to increase the awareness of young women and their physicians that a breast cancer diagnosis in this age group is not such a rare event, including during and right after pregnancy. Doctors should pay a particular attention to family history of breast and other cancers to evaluate the individual risk of the women and to early signs and symptoms that can be easily mistaken as benign findings.

The second invasive cancer in terms of frequency among young women in Geneva is thyroid. As observed in many affluent countries, incidence of thyroid cancer has markedly increased in the recent decades, although mortality has remained relatively low and stable [14]. This increase, attributed to a growing use of ultrasonography and other screening techniques in asymptomatic people, is now recognized as a major public health challenge [14]. As observed in other countries [15] we found incidence rates four times higher in women than in men. Although specific sex related factors that could explain this difference are not well documented, overdiagnosis as a result of increased surveillance is more prevalent among women [16], resulting in an increased rate of overtreatment. In Geneva 105 women diagnosed during 2014–2018, almost 70% of the total, were operated on for their thyroid cancer. As the survival is very high, most of these women will be monitored for many years and probably receive lifelong replacement therapy. The high human and financial costs of this practice are not always warranted. In South Korea, the country with the highest rates of thyroid cancer both among women and men [15], a call to end the practice of screening asymptomatic people and changes in guidelines for thyroid nodule examination have been associated with a decline in the rates of thyroid cancer detection while related mortality remains stable [17].

The third most common invasive cancer among women of this age group is melanoma of the skin, with a similar incidence rate to men. Incidence rates of melanoma have been steadily increasing since 1970 in Geneva as in other countries [18]. This has been the result of both increased detection due to greater awareness among patients and physicians, as well as a real increase in disease incidence as a consequence of greater exposure to sunlight, indoor tanning beds/lamps and sunbeds [19]. In the USA some authors estimate that melanoma will become the second most common cancer in the country by 2040 [20]. In Geneva we already observe this among men aged 20–49 years old. A skin melanoma is one of the most preventable forms of cancer: continued and sustained primary prevention efforts should be promoted particularly to children, adolescents and their parents to further improve UV radiation protection, along with secondary prevention activities to substantially reduce the burden of the disease in the population and health services.

Young adult women in Geneva also suffer from colorectal cancer, similarly to men. Recently, several high-income countries reported an increase in colorectal cancer among people younger than 50 years [21]. This finding motivated the recent recommendation to start screening at 45 years by the US Preventive Service Task Force [22]. Obesity, lack of physical activity, red and processed meat consumption are among the likely contributors to the rising trend [23].

Unlike most other registries, the Geneva cancer registry records pre-cancerous lesions. This allowed us to demonstrate that the incidence of non-invasive disease in young adult women is high as invasive disease. Non-invasive cancers are mostly diagnosed through screening that allows identifying asymptomatic conditions that would not be identified otherwise. This is true for breast cancer but especially for CIN3 lesions which were the most frequently diagnosed precancerous lesion among women in Geneva. The long and continuous use of screening is responsible for the very low rate of invasive cervical cancer morbidity and mortality in the canton (3.2/100′000 and 0.2/100′000 respectively) and probably also for lowering the age at which the disease is diagnosed in the process. However, the high rate of CIN 3 (121/100′000) implies an equally high burden of diagnostic investigations, treatments, and post-treatment care. Since 2006, Human Papilloma Virus (HPV) vaccination has been introduced in more than 100 countries [24]. A recent paper estimating the effect of routine HPV vaccination programme in England, showed a substantial reduction of both cervical cancer and CIN3 in vaccinated women. The benefit increased with younger age at vaccination [25]. To reduce the huge burden of disease related to HPV infection, there should be further efforts to assure high HPV coverage vaccination among young girls, but it will take another 15 years to materialise. Therefore, while increasing primary prevention coverage with the vaccine, women, should continue to be offered Pap test screening, with or without testing for HPV, and early treatment of precancerous lesions.

As noted, non-invasive lesions are mostly detected by breast or cervical cancer screening, not relevant for males, therefore we acknowledge that a comparison by gender of the occurrence of non-invasive conditions is challenging, leaving us with a rather small difference in rates of non-invasive melanoma.

## Conclusions

We found a substantial occurrence of cancer, both invasive and non-invasive, among women aged 20–49 years in Geneva. Depending on the type of cancer, this high burden is attributable to increased cancer risks, improved detection, including overdiagnosis, or a combination of the two. Young adult women are often unaware of these risks, experience delays in diagnosis and pay a high cost for these diagnoses and the treatments incurred. The findings of this study can help to increase awareness of women about their health issues, inform health professionals in recognizing and caring for such individuals, also through the development of personalized risk assessment and tailored screening, contribute to increasing primary prevention strategies, and encourage more research in this age group.

## Data Availability

The datasets used and/or analysed during the current study are available from the corresponding author on reasonable request. In compliance with data protection regulations, data are stored at the Geneva Cancer Registry, Geneva, Switzerland.
